# Effect of *Bifidobacterium breve* on the Intestinal Microbiota of Coeliac Children on a Gluten Free Diet: A Pilot Study

**DOI:** 10.3390/nu8100660

**Published:** 2016-10-22

**Authors:** Andrea Quagliariello, Irene Aloisio, Nicole Bozzi Cionci, Donata Luiselli, Giuseppe D’Auria, Llúcia Martinez-Priego, David Pérez-Villarroya, Tomaž Langerholc, Maša Primec, Dušanka Mičetić-Turk, Diana Di Gioia

**Affiliations:** 1 Laboratory of Molecular Anthropology, Centre for Genome Biology Department of Biological, Geological and Environmental Sciences (BiGeA), University of Bologna, via Selmi 3, Bologna 40126, Italy; andrea.quagliariello@unibo.it (A.Q.); donata.luiselli@unibo.it (D.L.); 2 Department of Agricultural Sciences, University of Bologna, viale Fanin 42, Bologna 40127, Italy; irene.aloisio@unibo.it (I.A.); nicole.bozzicionci@studio.unibo.it (N.B.C.); 3 Sequencing and Bioinformatics Service, Fundación para el Fomento de la Investigación Sanitaria y Biomédica de la Comunidad Valenciana (FISABIO-Salud Pública), Valencia 46020, Spain; dauria_giu@gva.es (G.D.); martinez_lucpri@gva.es (L.M.-P.); enhancertrap@gmail.com (D.P.-V.); 4 Department of Microbiology, Biochemistry, Molecular Biology and Biotechnology, Faculty of Agriculture and Life Sciences, University of Maribor, Pivola 10, Hoče 2311, Slovenia; tomaz.langerholc@um.si (T.L.); masa.primec@um.si (M.P.); 5 Department of Pediatrics, Faculty of Medicine, University of Maribor, Taborska ulica 8, Maribor 2000, Slovenia; dusanka.micetic@um.si

**Keywords:** coeliac disease, gluten free diet, probiotic, *Bifidobacterium breve*, intestinal microbiota, qPCR, next generation sequencing

## Abstract

Coeliac disease (CD) is associated with alterations of the intestinal microbiota. Although several *Bifidobacterium* strains showed anti-inflammatory activity and prevention of toxic gliadin peptides generation in vitro, few data are available on their efficacy when administered to CD subjects. This study evaluated the effect of administration for three months of a food supplement based on two *Bifidobacterium breve* strains (B632 and BR03) to restore the gut microbial balance in coeliac children on a gluten free diet (GFD). Microbial DNA was extracted from faeces of 40 coeliac children before and after probiotic or placebo administration and 16 healthy children (Control group). Sequencing of the amplified V3-V4 hypervariable region of 16S rRNA gene as well as qPCR of *Bidobacterium* spp., *Lactobacillus* spp., *Bacteroides fragilis* group *Clostridium* *sensu stricto* and enterobacteria were performed. The comparison between CD subjects and Control group revealed an alteration in the intestinal microbial composition of coeliacs mainly characterized by a reduction of the *Firmicutes/Bacteroidetes* ratio, of *Actinobacteria* and *Euryarchaeota*. Regarding the effects of the probiotic, an increase of *Actinobacteria* was found as well as a re-establishment of the physiological *Firmicutes/Bacteroidetes* ratio. Therefore, a three-month administration of *B. breve* strains helps in restoring the healthy percentage of main microbial components.

## 1. Introduction

Coeliac disease (CD) is a chronic gastrointestinal tract disorder showing damages at the small intestine, which are hypothetically linked to an autoimmune response caused by gluten ingestion in genetically predisposed subjects. CD in Europe and North America is estimated to affect about 1% of the population, although its incidence in Western countries is increasing in the last decades [1,2]. CD is usually chronic but the lifelong adherence to a gluten-free diet (GFD) keeps the disease under control: the small intestine returns to its physiological condition and subsequent tests for CD specific autoantibodies are negative [3,4]. Even if the adherence to a GFD is the only effective solution against CD, patients risk suffering from an unbalanced nutritional intake and difficulties to adhere to the strict GFD are frequently reported.

The gut microbiota has a very close relation with the host contributing to the normal human physiology. It can provide a barrier for colonization of pathogens, synthesize vitamins and other beneficial compounds and stimulate the immune system. Environmental factors can lead to a disturbance of the microbiota composition, disrupting microbiota-host mutualism and shifting from a condition of homeostasis to a disease-associated profile [5]. In the last decade, CD has been associated to an altered composition of the intestinal microbiota even though studies reported in literature show that there is not a characteristic “coeliac intestinal microbiota” [6]. Some authors evidenced an intestinal dysbiosis in CD patients with active disease characterized by a remarkable reduction in Gram positive bacterial population in duodenal and faecal specimens facilitating the colonization of potentially harmful Gram negative bacteria within the mucosal surface of CD patients [7,8,9]. In particular, data obtained from duodenal biopsies revealed a reduction in the number of bifidobacteria [10] and changes in species distribution within the *Bifidobacterium* genus have been evidenced by Denaturing Gradient Gel Electrophoresis (DGGE) [11]. Moreover, symptom free CD patients adherent to a GFD at least for two years did not completely restore the microbiota composition and this condition can lead to a different metabolomics profile [9]. Bacteria belonging to the *Bifidobacterium* genus are well known for their health promoting properties and for their capability of stimulating cells to produce immune molecules and modulating the physiology of gut-associated lymphoid tissue (GALT) [12]. In particular, in vitro studies have been focused on the capability of bifidobacteria to increase the IL-10 secretion when co-incubated with mononuclear cells and faecal samples from CD patients [13]. Moreover, a *Bifidobacterium lactis* strain and a probiotic product containing *Lactobacillus* and *Bifidobacterium* strains resulted effective in reducing gliadin induced epithelial permeability through prevention of the toxic gliadin peptide generation during in vitro digestion [14,15,16].

Despite the encouraging data on the potential of probiotic strains, particularly bifidobacteria, in vivo studies in patients with CD remain still very scarce. Until now, only few studies have taken into account the direct administration of bifidobacteria in subjects affected by CD. Smecuol et al. [17] studied the effects of *Bifidobacterium infantis natren life start* strain in untreated CD patients or rather on a gluten containing diet. Authors found that *Bifidobacterium* administration may alleviate symptoms of untreated CD but it could not modify intestinal permeability. A second study [18] evaluated the administration of *Bifidobacterium longum* CECT 7347 in children on a GFD with newly diagnosed CD and it revealed a reduction of CD3+ T lymphocytes and TNF-α due to probiotic ingestion. To date, no studies on CD considered the administration of *Bifidobacterium breve* strains although this species has proven very successful in several paediatric trials regarding necrotizing enterocolitis, immunodeficiency and constipation [19,20,21].

This work is aimed at the assessment of the impact of the administration of two *Bifidobacterium breve* strains on the gut microbiota composition of coeliac patients compliant to a GFD and, at the same time, it evaluates the difference in the intestinal colonization of coeliac subjects on a GFD for several years with respect to healthy subjects.

## 2. Materials and Methods

### 2.1. Study Design and Samples Collection

The study was a double-blinded placebo controlled intervention including 40 patients affected by CD and 16 healthy children for the Control group recruited at a single centre, Department of Paediatrics, University Clinical Center Maribor in a period from October 2013 to June 2014. Children with CD, aged between 1 and 19 years, were positive to serologic markers for CD and positive for small bowel biopsy, according to European Society for Paedriatric Gastroenterology Hepatology and Nutrition (ESPGHAN) criteria for CD [22]. More details about patients (Table S1) and inclusion/exclusion criteria of the recruiting process are available in Klemenak et al. [23]. The study was registered at https://www.clinicaltrials.gov (registration number: NCT02244047). Patients affected by CD have been randomly allocated into two groups: 20 in the Probiotic group and 20 in the Placebo group. The Probiotic group of patients received an experimental formulation containing *B. breve* for three months and the Placebo group received a placebo formulation for the same duration. Probiotic formulation was a mixture of 2 strains, *B. breve* BR03 (DSM 16604) and *B. breve* B632 (DSM 24706) (1:1), administered as lyophilized powder in a daily dosage of 10^9^ Colony Forming Units (CFU) of each strain. Placebo was prepared with the same excipients without probiotic strains using an identical form of package. Each package of 2 g powder was mixed with fluids and ingested in the morning breakfast for three months.

Faecal samples of CD patients were collected twice, on enrolment (T0) and at the end of intervention with probiotic/placebo (T1). Members of Control group were sampled only once. Faecal samples were frozen immediately after collection at −80 °C, in numbered screw-capped plastic containers, until they were processed for DNA extraction. Researchers carrying out DNA extraction and molecular analyses (qPCR and sequencing) were blind to the group identity of patients (Control, Probiotic or Placebo group).

### 2.2. DNA Extraction from Faecal Samples

DNA was extracted from 200 mg of faeces (preserved at −80 °C after collection) were used using the QIAamp DNA Stool Mini Kit (Qiagen, West Sussex, UK) with a slight modification of the protocol: An additional incubation at 95 °C for 10 min of the stool sample with the lysis buffer was added to improve the bacterial cell rupture [24]. Extracted DNA was stored at −80 °C. The purity of extracted DNA was determined by measuring the ratio of the absorbance at 260 and 280 nm (Infinite^®^200 PRO NanoQuant, Tecan, Mannedorf, Switzerland) and the concentration was estimated by Qubit^®^ 3.0 Fluorometer (Invitrogen, Life Technologies, Van Allen Way, Carlsbad, CA, USA).

### 2.3. Preparation of DNA Libraries for Illumina MiSeq Sequencing

**Figure 1:**
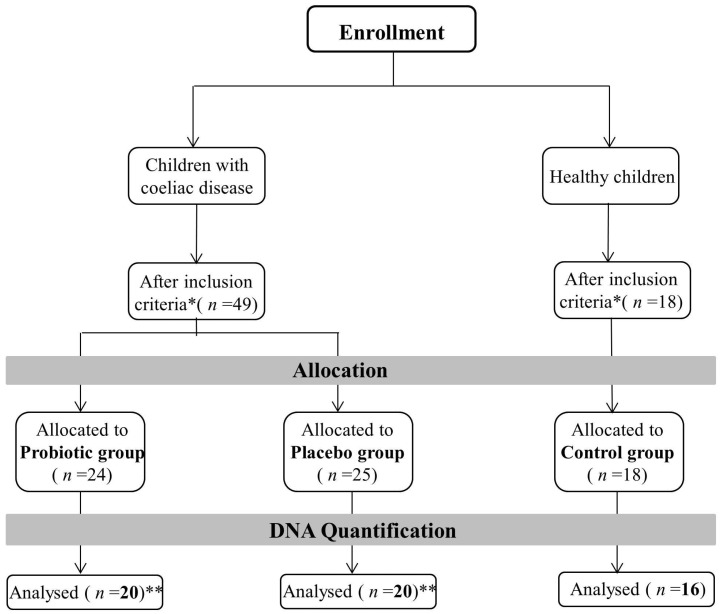
Summary of inclusion/exclusion criteria adopted for samples selection. Samples who showed the right DNA quantification level have been sequenced. * Inclusion criteria are summarized in Klemenak et al. [23]. ** Analysis was performed at the beginning of the study (T0) and after 3 months of treatment (T1).

The sample subjected to sequencing belonged to the following groups: 20 Probiotic group T0, 20 Probiotic group T1, 20 Placebo group T0, 20 Placebo group T1 and 16 Control group (Figure 1).

They were processed to amplify and sequence the V3-V4 region of the 16S rRNA gene. The amplicons, approximately 460 bp in length, were generated using the forward and reverse primers, respectively: 5′ TCGTCGGCAGCGTCAGATGTGTATAAGAGACAGCCTACGGGNGGCWGCAG 3′, 5′ GTCTCGTGGGCTCGGAGATGTGTATAAGAGACAGGACTACHVGGGTATCTAATCC 3′, already used in [25].

Each 25 µL PCR reaction contained 12.5 µL of HiFi HotStart ReadyMix (KAPA Biosystems, Woburn, MA, USA), 5 µL of each primer (0.2 µM) and microbial DNA (5 ng/µL). PCR amplification was performed using the following program: Heated lid at 110 °C, 95 °C for 3 min followed by 25 cycles at 95 °C for 30 s, 55 °C for 30 s, and 72 °C for 30 s, followed by a final elongation step at 72 °C for 5 min. PCR products were cleaned using the AMPure beads XP purification system (Beckman Coulter, UK) following Illumina 16S Ribosomal RNA Gene Amplicon instructions. Illumina sequencing adapters and dual-index barcodes were added to amplicons using the Nextera XT index kit (Illumina, San Diego, CA, USA). The following program was utilized for the second PCR amplification: 95 °C for 3 min followed by 8 cycles of 95 °C for 30 s, 55 °C for 30 s and 72 °C for 30 s and a final elongation at 72 °C for 5 min. A further clean up protocol using AMPure beads XP purification system (Beckman Coulter, UK) is performed. Amplicons were quantified using the Qubit^®^ 2.0 Fluorometer (Invitrogen) and pooled equimolar to 4 nM. The pool was denatured with 0.2 M NaOH, further dilution with hybridization buffer to 20 pM and combined with denatured 30% PhiX. Samples were sequenced on the Illumina MiSeq platform at the Fundacion FISABIO (Valencia, Spain) facility using a 2 × 300 nucleotide paired reads protocol. Sequencing raw data were deposited at European Nucleotide Archive (ENA) and received the following ID: PRJEB14943.

### 2.4. Bioinformatics and Statistical Analyses of NGS Experiment

Several bioinformatics pipelines have been used to analyse the amount of data produced during this project. The first step of analysis was represented by quality controls of the generated raw data, which are essential to be confident of the quality of the experimental results. For this purpose, the FastQC 0.11.4 software (Babraham Bioinformatics) was used for a rapid visualization of sequences quality, then with the prinseq-lite.pl script sequences have been trimmed according to various quality criteria: first of all sequences with less than 50 bp were eliminated, then remaining reads were analyzed with a sliding-window approach of 20 bp, within this range each sequence with a mean quality lower than 20 was removed [26].

After that, the fastq-join tool from the ea-tools suite [27] was used to join forward and reverse sequences. The last quality control step was represented by the elimination of chimeric sequences using the Usearch tool (http://drive5.com/usearch/). Once high-quality double-stranded reads were obtained, they were aligned to the 16S reference sequences database at the RDP database project to identify the microbial community with the RDP classifier tool [28]. RDP classifier outputs have been then processed through several R software packages, such as *vegan*, *reshape2*, *RDPutils* and *phyloseq* in order to estimate various biodiversity indexes and to perform the principle statistics analyses on taxonomic profiles. Finally, data have been normalized and the function exactTest() of the *edgeR* package was used to evaluate the effective microbial differentiation among the studied groups [29].

### 2.5. Absolute Quantification of Selected Microbial Groups Using Quantitative PCR (qPCR)

Quantification of selected microbial groups i.e., *Bidobacterium* spp., *Lactobacillus* spp., *Bacteroides fragilis* group (comprising the species *B. fragilis*, *B. distasonis*, *B. ovatus*, *B. thetaiotaomicron*, *B. vulgatus*), *Clostridium* *sensu stricto* or cluster I and total enterobacteria, was carried out with real-time PCR on DNA extracted from faecal samples. The assays were performed with a 20 μL PCR amplification mixture containing 10 μL of Fast SYBR^®^ Green Master Mix (Applied Biosystems, Foster City, CA, USA), optimized concentrations of primers (Table 1 and Table 2), H_2_O molecular grade and 2 μL DNA extracted from faecal samples at a concentration of 2.5 ng/μL for all the assays. The primer concentrations were optimized through primer optimization matrices in a 48-well plate and evaluating the best Ct/Rn ratio. The different primers were also checked for their specificity using the database similarity search program nucleotide-nucleotide BLAST [30]. Moreover, to determine the specificity of amplification, analysis of product melting curve was performed after the last cycle of each amplification. The data obtained from the amplification were then transformed to obtain the number of bacterial Log CFU/g faeces according to the rRNA copy number available at the rRNA copy number database [31]. Standard curves were constructed using 16S rRNA PCR product of type strains of each target microorganism. PCR products were purified with a commercial kit DNA purification system (NucleoSpin^®^ Extract II kit, MACHEREY-NAGEL GmbH & Co. KG, Duren, Germany) and the concentration measured at 260 nm. Serial dilutions were performed and 10^2^, 10^3^, 10^4^, 10^5^, 10^6^, and 10^7^ copies of the gene per reaction and were used for calibration.

Data of microbial counts were subjected to *T*-test in order to evidence significant differences between treated and Control group of subjects.

## 3. Results

### 3.1. Metagenomic Analysis

The V3-V4 region of 16S rDNA gene was sequenced from 96 DNA samples using the Illumina MiSeq platform. A total dataset of 4,348,432 filtered high-quality joined reads (excluding the undetermined sequences) was thus generated, about 46,259 sequences per sample, with a mean quality between 30 and 35. Two samples were excluded from the whole dataset because they did not pass the established quality threshold.

**Figure 2:**
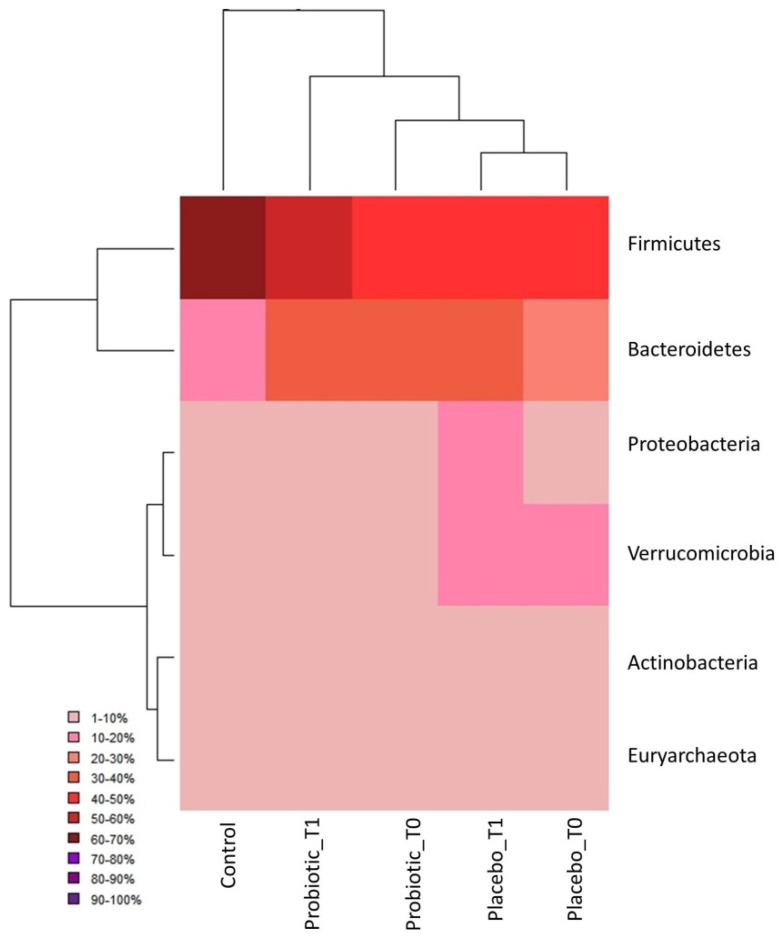
Hierarchically clustered heat map: Sample groups are reported in column, while phyla are reported in row.

Massive sequencing revealed the presence of six phyla (five belonging to Bacteria and one to Archaea) with a relative abundance higher than 1%: *Firmicutes, Bacteroidetes, Proteobacteria, Actinobacteria, Verrucomicrobia* and *Euryarchaeota*. The obtained phyla had a different distribution among the five groups of examined subjects as highlighted in the heat map (Figure 2), in particular in the *Firmicutes* and *Bacteroidetes* phyla.

In particular, the *Firmicutes* phylum showed the highest representativeness in the Control group (accounting for 60%–70% of the total microbial community), whereas it reached 50%–60% in Probiotic T1 and 40%–50% in the rest of CD patients (Probiotic T0, Placebo T0, and Placebo T1 groups).

On the other hand, the *Bacteroidetes* phylum was more abundant within CD subjects (20%–40%) than in the Control group subjects (10%–20%). The other phyla were more evenly distributed among groups, with the only difference for *Proteobacteria* and *Verrucomicrobia* that were more represented in the Placebo group (~10%–20%). Moreover, the hierarchical cluster analysis combined with the heat map pointed out that the Probiotic T1 group occupied an intermediate position between the Control group and the rest of CD individuals, being thus considered as an outlier with respect to the other disease clusters because of its closer relationship with control subjects.

**Figure 3:**
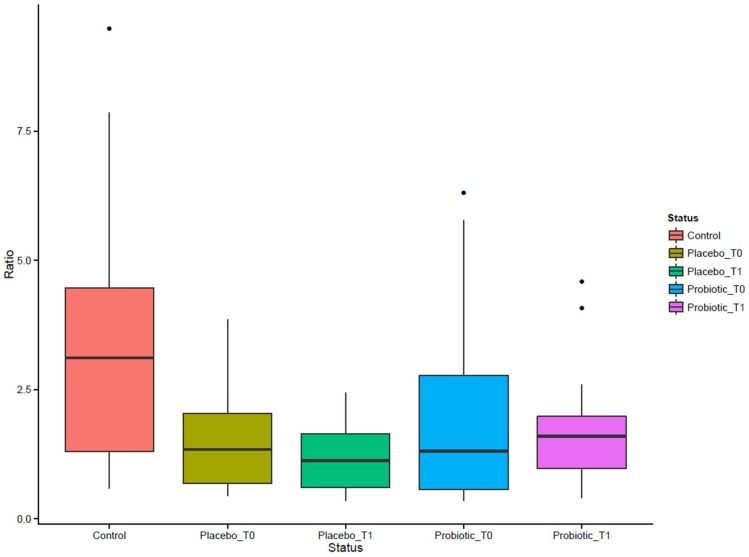
The *Firmicutes/Bacteroidetes* ratio.

From the comparison between the CD subjects and the Control group microbiota emerged a marked difference in the ratio of *Firmicutes/Bacteroidetes.* Figure 3 shows values of ratio *Firmicutes/Bacteroidetes* calculated for each group of subjects. CD subjects had a ratio values lower than the Control group thus meaning a high proportion of *Bacteroidetes* (Gram negative) with respect to *Firmicutes* (Gram positive). The administration of the probiotic for three months was found to increase the ratio value due to the higher level of *Firmicutes* phyla than *Bacteroidetes*.

**Figure 4:**
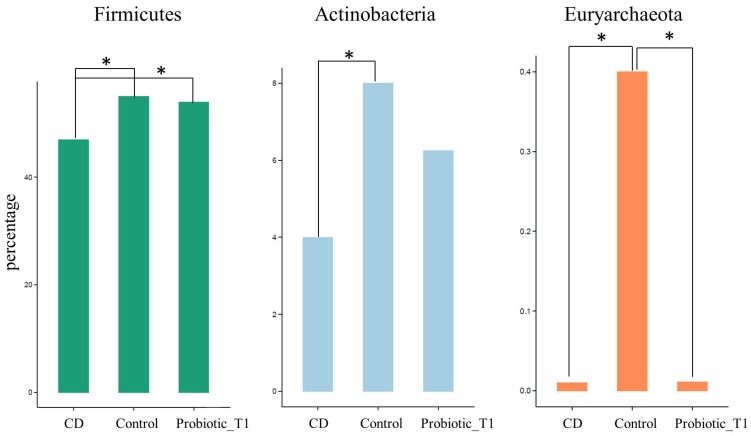
Relative abundance of the three phyla that show a statistical significance difference among CD, Control and Probiotic T1. CD group is composed of Probiotic T0, Placebo T0 and Placebo T1 samples. The * indicates *p* < 0.01. Supporting information on relative abundance and *p*-values is found in Tables S2 and S3.

Following the data normalization procedure and assignation of statistical significance described in Material and Method, several comparisons between pair of groups were performed in order to identify which phyla could distinguish the microbiota of Control group from that of CD patients not assuming the probiotic formulation, and from Probiotic T1 (Figure 4).

**Figure 5:**
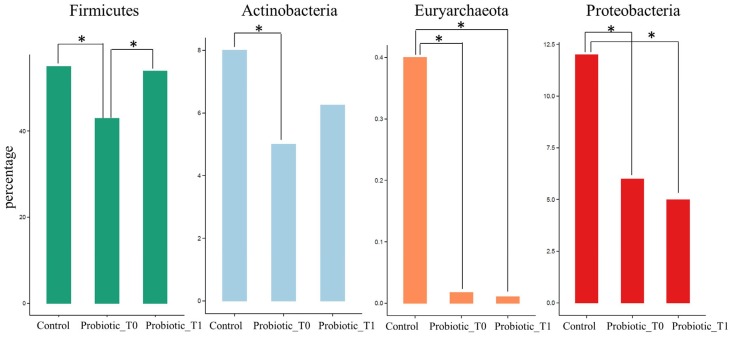
Significant differences in phyla relative abundance among Control, Probiotic T0 and Probiotic T1 groups. The * indicates *p* < 0.01. Supporting information on relative abundance and *p*-values is found in Tables S4 and S5.

Statistical analyses confirmed that *Firmicutes* were significantly lower in CD subjects not receiving the probiotic formulation compared to Controls and Probiotic group (*p* < 0.01). Similar results were found for *Actinobacteria* that were underrepresented in the CD group and increased after the administration of bifidobacteria, although not reaching the abundance found in the controls. A further discernment regarded the *Euryarchaeota* phylum belonging to *Archaea* that was almost exclusively present in the Control group. The same analysis was repeated comparing the microbial composition of the Control group with the Probiotic groups before and after the probiotic administration (respectively Probiotic T0 and Probiotic T1) (Figure 5). The comparison highlighted an increase in the relative abundance of *Firmicutes* (*p* < 0.01) and *Actinobacteria*, due to the effect of probiotic administration. On the other hand it was possible to observe a slight decrease of the abundance of *Proteobacteria* while the *Euryarchaeota* phylum kept unchanged after the treatment.

**Figure 6:**
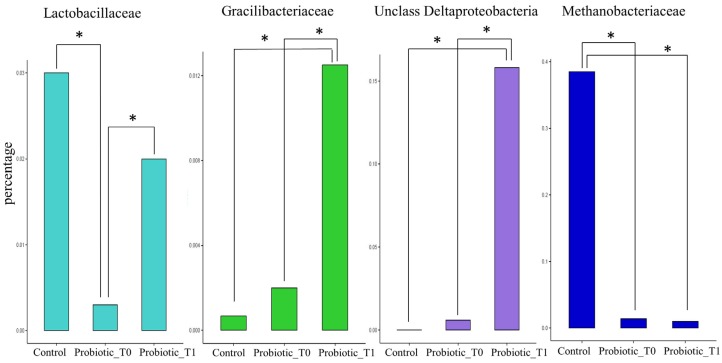
Statistical significant differences in families relative abundance among Control, Probiotic T0 and Probiotic T1 groups. The * indicates *p* < 0.01. Supporting information on relative abundance and *p*-values is found in Tables S6 and S7.

The same comparison was carried out at the family taxonomic level. Within the *Firmicutes* phylum, two families, which are poorly represented in the Probiotic T0 group, showed instead a higher level in both the Probiotic T1 and the Control groups: *Lactobacillaceae* and *Gracilibacteraceae*. In particular, both bacterial families showed a significant different abundance between Probiotic T0 and Probiotic T1 groups, whereas no differences were observed between Probiotic T1 and Control groups. In contrast, Probiotic T1 subjects demonstrated a high percentage of unclassified *Deltaproteobacteria* families. Moreover, this analysis enabled identifying the *Methanobacteriaceae* family as almost exclusively present within the Control group (Figure 6).

**Figure 7:**
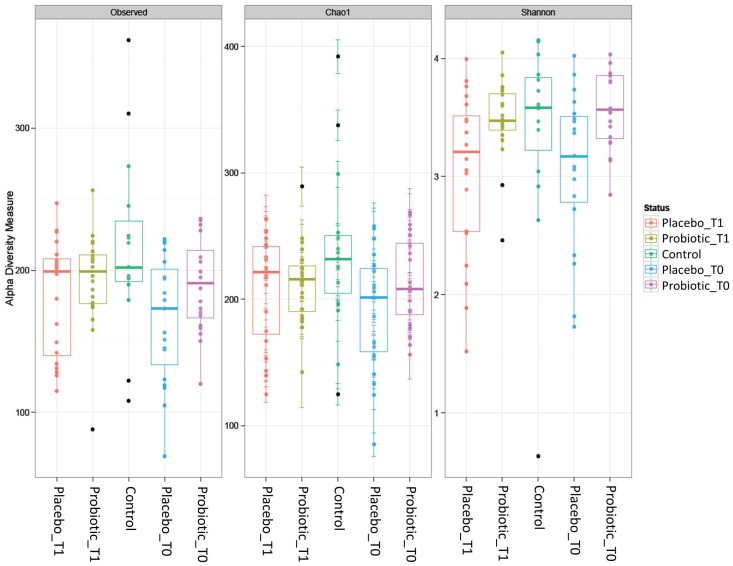
Alpha diversity indices among the studied groups.

The α-diversity indices (Observed, Chao1 and Shannon) were computed for all Operational Taxonomic Units (OTUs) founded in the five groups of samples as reported in Figure 7. No significant changes in OTUs composition among the studied groups were observed. Particularly, the observed raw biodiversity, as well as the Chao1 index, were slightly higher in the control samples than in all the other groups, but the differences were not significant. Even the Shannon index indicated similar trends among all groups, with a mean value of about 3. This similarity among groups was further confirmed by the application of Wilcoxon test on these indices, which indicated the totally absence of significant differences.

### 3.2. Quantification of Selected Microbial Groups in Faecal Samples

qPCR analysis was carried out in order to obtain the absolute quantification of selected microbial groups as a supplementary information able to complete the microbial profile of the examined subjects. Faecal samples were collected and DNA extracted at two sampling times for CD subjects, on enrolment (Probiotic T0 + Placebo T0) and at the end of the three months intervention with probiotic or placebo (T1), and once for healthy individuals (control group). Quantification regarded specific microbial genera typical of the human gut, *Bifidobacterium* spp., *Clostridium* *sensu stricto*, *Bacteroides fragilis* group (comprising the most abundant species in human, i.e., *B. fragilis*, *B. distasonis*, *B. ovatus*, *B. thetaiotaomicron*, and *B. vulgatus*), and larger microbial group, *Lactobacillus* group, which include *Lactobacillus*, *Pediococcus*, *Leuconostoc* and *Weisella* species, and total enterobacteria comprehensives of a larger number of gram-negative intestinal bacteria. The average microbial counts obtained are shown in Table 3.

**Figure 8:**
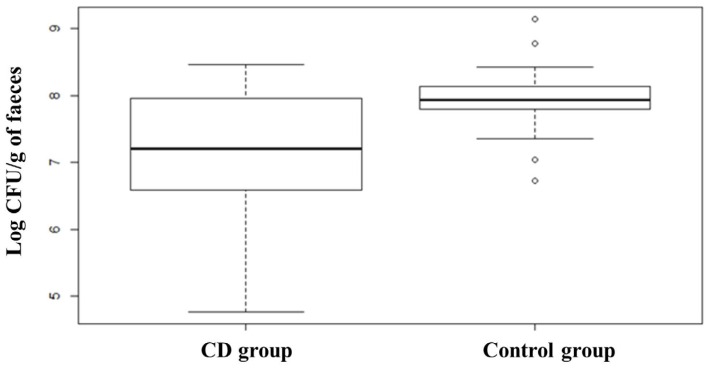
Box plots showing qPCR analysis of *Lactobacillus* group expressed in log CFU per gram of faecal sample relative to CD group and Control group. CD group is composed of Probiotic T0, Placebo T0 and Placebo T1 samples. Statistical difference between the two groups (*p*-values of < 0.01).

**Figure 9:**
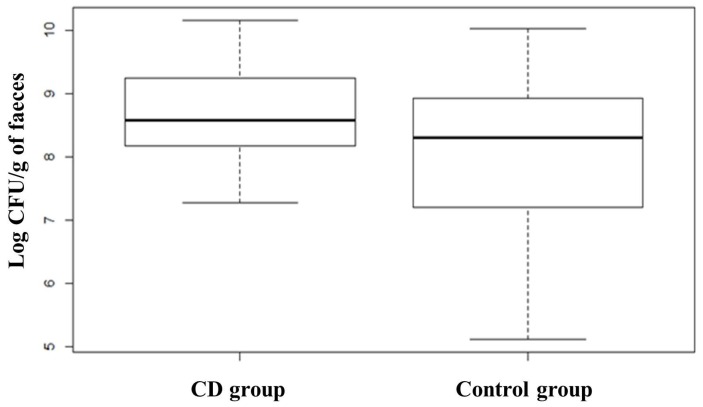
Box plots showing qPCR analysis of *Bacteroides fragilis* group expressed in log CFU per gram of faecal sample relative to CD group and Control group. CD group is composed of Probiotic T0, Placebo T0 and Placebo T1 samples. Statistical difference between the two groups (*p*-values of < 0.01).

Quantification of *Bifidobacterium* spp. evidenced a slightly higher value in the subjects affected by CD at T0 with respect to the control group, although this difference was not significant. The comparison between subjects belonging to the probiotic group before and after the treatment showed that the administration of the probiotic formula containing *Bifidobacterium breve* led to a slight increase of bifidobacteria counts from 7.64 ± 1.01 to 8.06 ± 0.98 Log CFU/g of faeces. *Lactobacillus* spp. group analysis revealed that healthy subjects (Control group) had a higher presence of members of this group compared to CD patients, which on the contrary, showed a great heterogeneity in the distribution (Figure 8). ANOVA test revealed that the difference was statistically significant (*p* < 0.01). The opposite trend was found for members of *Bacteroides fragilis* group showing a higher median in CD subjects compared to healthy subjects, as shown in the box plot (Figure 9). The box plot relative to healthy subjects is shorter than the other one and it also shows a higher median value but a narrower distribution of the data. ANOVA test revealed that the difference is statistically significant (*p* < 0.01). CD patients showed more than 8.70 Log CFU/g of faeces of *Bacteroides fragilis* group bacteria. No significant differences were recorded concerning changes in the levels of *Bacteroides* due to treatment with probiotics.

**Figure 10:**
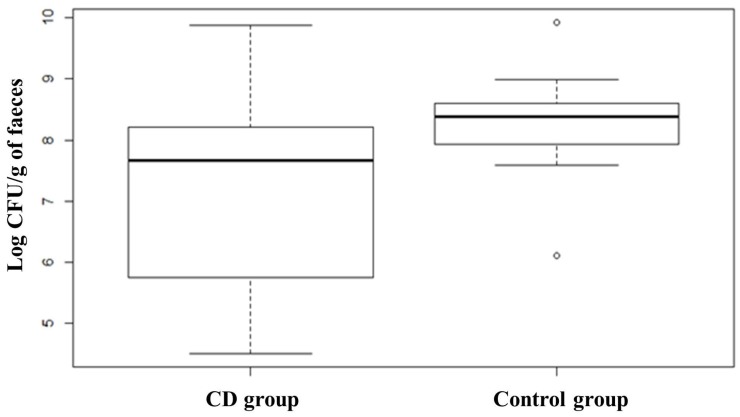
Box plots showing qPCR analysis of total enterobacteria expressed in log CFU per gram of faecal sample relative to CD group and Control group. CD group is composed of Probiotic T0, Placebo T0 and Placebo T1 samples. Statistical difference between the two groups (*p*-values of < 0.01).

With regard to enterobacteria, they were more abundant in the control group compared to CD patients: 8.29 ± 0.80 and 7.10 ± 1.24 CFU/g, respectively. This trend can also be outlined from the graphs reported in Figure 10, which clearly shows that the median value of control group is higher than CD groups, the latter showing a lower level of enterobacteria with a higher heterogeneity. Furthermore, after the three months of probiotic treatment it was possible to observe a decreased level of enterobacteria in Probiotic T1 (Figure 10 and Table 3). Regarding *Clostridium* *sensu stricto*, its quantification was lower than the other microbial groups (values from 5.83 to 6.19 Log CFU/g of faeces). No statistical differences resulted from the comparison between control and CD patients and between Probiotic and Placebo groups.

## 4. Discussion

This work was focused on the characterization of the major changes occurring in the intestinal microbiota of CD patients on a GFD and on the evaluation of the effects that the administration of two *B. breve* strains (B632 and BR03) may have on these patients.

In the last few years a particular attention has been paid on the correlation between gut microbiota composition and CD. Several studies demonstrated an increase in gram-negative bacteria, mainly belonging to the *Bacteroidetes* phylum, at the expense of microorganisms of the *Actinobatceria* and *Firmicutes* phyla in subjects with active disease [7,8,10], in agreement with the results registered in other chronic inflammatory gastrointestinal diseases such as inflammatory bowel disease [37]. However, these differences did not allow identifying a coeliac microbiota signature directly linked to CD [6].

Although data regarding the health promoting properties of bifidobacteria and more in general of probiotic microorganisms are well documented, their role in the treatment of CD has been scarcely investigated. The two strains administered in this work, *B. breve* B632 and BR03, are known to possess anti-inflammatory activity stimulating intestinal cells in vitro to produce IL-6 and IL-10, respectively [38,39] and have been previously characterized for safety issues such as the absence of transmissible antibiotic resistance traits and toxicity towards gut epithelial cells. In addition, the two strains in combination showed a great capability of colonizing the gut of healthy children [40]. In relation to CD, a preliminary important outcome obtained from the administration of the described probiotic formulation to CD patients was the reduction of pro-inflammatory cytokine TNF-α in blood samples of CD subjects on a GFD after three months of treatment, as reported in Klemenak et al. [23].

The first interesting evidence that emerges from the present study is the absence of a severe intestinal dysbiosis in CD patients on a GFD diet, as shown by the comparison of the α-diversity similarity indices and the absence of statistically significant differences in OTU variability in the analysed cohort of CD patients with respect to the Control group. On the contrary, literature data related to active disease patients non-adherent to a GFD showed the presence of extensive changes in the microbial composition [8]. Therefore, the strict adherence to the GFD partially recovers the intestinal equilibrium status.

However, the results obtained in this study showed a significant quantitative difference in some microbial groups by qPCR and by metagenomic analysis in CD patients with respect to the Control group. The elaboration of the microbial relative abundance data obtained by Illumina MiSeq sequencing were able to clearly separate CD subjects from the Control group ones. The lower number of *Bacteroidetes* phylum in CD patients with respect to the Control group was supported by *B. fragilis* group quantification by qPCR and it is consistent with the results of another study on CD patients on GFD [7]. The obtained results are also in agreement with the observation that CD subjects present an imbalance in the *Firmicutes*/*Bacteroidetes* ratio, usually lower in CD patients, and this ratio is not completely restored in patients under a GFD [41]. Moreover, the probiotic administration induced an evident increase of *Firmicutes* abundance while maintaining a similar percentage of *Bacteroidetes*, thus resulting in a higher value of the *Firmicutes*/*Bacteroidetes* ratio. In addition, the Control group microbiota seems to be characterized by a higher percentage of *Actinobacteria* and *Euryarchaeota*. The association between CD disease status and a lower presence of *Actinobacteria* has already been described [42]. Particularly interesting, although not yet described in the literature, is the result regarding the *Euryarchaeota* phylum, which is highly represented in the Control group, but almost absent in the coeliac subjects. The same applies for the *Methanobacteriaceae* family. This evidence could conceivably be linked to differences in the dietary habits of the two groups of subjects. Recent research works focused on *Euryarchaeota* highlighted their ability to promote polysaccharide degradation and absorption of fatty acids, thus they seem to play a role in energy extraction from degradation of organic compounds [43]. Grain is the most common source of polysaccharides in modern human populations, thus the important reduction of archaea microorganisms within coeliac group on GFD is linked to their different nutritional status, in particular to the compliance of the GFD and the consequent lower polysaccharide intake.

Focusing on the effects of the administration of the *B. breve* strains on microbial composition, an increase of members of the *Actinobacteria* phylum (NGS) and bifidobacteria (qPCR) have been detected in the CD subjects after three months of probiotic supplementation, although the increase was not statistically significant. One of the possible reasons could be the short duration of the treatment, furthermore it is already known that, after the weaning period, the microbiota is resilient to changes [44]. The treatment with the *B. breve* strains has therefore not caused major changes at the level of the genus or phylum to which the probiotic belongs, as it might have been expected, but the intake of the probiotic has nevertheless acted as a “trigger” element for the increase of *Firmicutes* and the restoration of the physiological *Firmicutes/Bacteroidetes* ratio. By reaching the microbial family level of analysis, it was possible to get more details on the effect of probiotic administration, allowing to reach the conclusion that two *Firmicutes* families (*Lactobacillaceae* and *Gracilibacteraceae*) changed their relative abundances upon probiotic treatment (Probiotic T1 group), particularly *Lactobacillaceae* that reached almost the values that characterized the Control group. Other studies have also observed a lower presence of *Lactobacillaceae* in CD patients, indicating a close relationship between this pathological condition and the bacterial family [9]. This means that the probiotic has restored the normal amount of *Lactobacillaceae* members belonging to these families within the treated individuals. It remains to be explained why the administration of such a *Bifidobacterium* strain have affected *Lactobacillaceae* species. This could be related to a high ability of *Bifidobacterium* to deep influence gut microflora composition, by enhancing the blooming of some species and antagonizing others probably by the effect of the production of metabolites such as acetic acid [45]. In particular, there are evidences that *Bifidobacterium* support *Lactobacillaceae* development [46]. Moreover, it is highly probable that the decrease of TNF-α observed within treated individuals is closely linked to the increase of lactobacilli, with their anti-inflammatory function promoted by the administration of *Bifidobacterium* [47,48].

## 5. Conclusions

In conclusion, the present study demonstrated that three months administration of *B. breve* strains could make the intestinal microbiota of coeliac patients more similar to that of healthy individuals, restoring the abundance of some microbial communities that characterize the typical physiological condition.

## Figures and Tables

**Table 1:** Primer sequences and qPCR conditions used in the different assays.

Target Microorganisms	Primer Sequences (5′-3′)	Amplicon Length (bp)	References	Annealing Temperature
*Bifidobacterium* spp.		243	[32]	55 °C
BifTOT-F	TCGCGTCYGGTGTGAAAG			
BifTOT-R	CCACATCCAGCRTCCAC			
*Lactobacillus* spp.		349	[33]	60 °C
Lac-F	GCAGCAGTAGGGAATCTTCCA			
Lac-R	GCATTYCACCGCTACACATG			
*Bacteroides fragilis* group		92	[34]	58 °C
Bfra-F	CGGAGGATCCGAGCGTTA			
Bfra-R	CCGCAAACTTTCACAACTGACTTA			
Enterobacteria		195	[35]	63 °C
Eco 1457F	CATTGACGTTACCCGCAGAAGAAGC			
Eco 1652R	CTCTACGAGACTCAAGCTGC			
*Clostridium cluster* I		232	[36]	52 °C
CI-F1	TACCHRAGGAGGAAGCCAC			
CI-F2	GTTCTTCCTAATCTCTACGCAT			

**Table 2:** qPCR amplification protocols and primer concentrations.

Target Microorganisms	Initial Denaturation	Denaturation	Annealing	Cycles	Fw nM	Rev nM
*Bifidobacterium* spp. BifTOT F/BifTOT-R	95 °C, 20 s	95 °C–30 s	60 °C–30 s	40	200	300
*Lactobacillus* spp. LAC-F/LAC-R	95 °C, 20 s	95 °C–30 s	63.5 °C–30 s	40	200	200
*Bacteroides fragilis* group Bfra-F/Bfra-R	95 °C, 20 s	95 °C–30 s	60 °C–30 s	40	300	300
Enterobacteria Eco-F/Eco-R	95 °C, 20 s	95 °C–30 s	60 °C–30 s	40	400	400
*Clostridium cluster* I CI-F1/CI-F2	95 °C, 20 s	95 °C–30 s	60 °C–30 s	40	200	200

Fw = Primer Forward, Rev = Primer Reverse.

**Table 3:** Mean counts of different microbial groups analysed in stool samples expressed as Log CFU/g of faeces.

Target	Log No. CFU/g of Faeces				
	Probiotic Group		Placebo Group		Control Group
	T0	T1	T0	T1	T0
*Bifidobacterium* spp.	7.64 ± 1.01	8.06 ± 0.98	7.82 ± 0.80	7.74 ± 0.73	7.26 ± 0.92
*Lactobacillus* spp.	6.87 ± 1.08	6.92 ± 0.95	7.21 ± 0.80	7.04 ± 0.97	7.84 ± 0.58
*B. fragilis* group	8.73 ± 0.79	8.71 ± 0.77	8.74 ± 0.76	8.84 ± 1.03	7.46 ± 1.47
Enterobacteria	7.10 ± 1.24	6.75 ± 1.29	7.25 ± 1.81	7.63 ± 1.48	8.29 ± 0.80
*Clostridium* *sensu stricto*	5.97 ± 0.96	5.83 ± 0.87	6.17 ± 0.95	6.19 ± 0.81	5.86 ± 0.80

## Supplementary Materials

The following are available online at http://www.mdpi.com/2072-6643/8/10/660/s1, Table S1: Description of the study groups at the beginning of intervention, Table S2: *p*-values among groups. Table S3: Mean values of relative abundance with standard deviation, Table S4: *p*-values among groups, Table S5: Mean values of relative abundance with standard deviation, Table S6: *p*-values among groups, Table S7: Mean values of relative abundance with standard deviation.
