# Supplementary material for: Effect of Bifidobacterium breve on the Intestinal Microbiota of Coeliac Children on a Gluten Free Diet: A Pilot Study
Source: Nutrients. 2016 Oct 22;8(10):660. doi: 10.3390/nu8100660 (PMC5084046; doi:10.3390/nu8100660)
Supplement: Supplementary file 1 [file nutrients-08-00660-s001.pdf]

Supplementary Materials: Effect of *Bifidobacterium breve* on the Intestinal Microbiota of Coeliac Children on a Gluten Free Diet: A Pilot Study

Andrea Quagliariello, Irene Aloisio, Nicole Bozzi Cionci, Donata Luiselli, Giuseppe D'Auria, Llúcia Martinez-Priego, David Pérez-Villarroya, Tomaž Langerholc, Maša Primec, Dušanka Mičetić-Turk and Diana Di Gioia

Table S1. Description of the study groups at the beginning of intervention.

	Placebo Group	Probiotic Group	Control Group
**No. patiens**	20	20	16
**Sex (M/F)**	8/12	6/14	7/9
**Age (year)**	10.53 ± 3.87	10.23 ± 3.18	8.80 ± 5.12
**Time on GFD (year)**	7.16 ± 4.98	5.43 ± 3.40	-
**Compliance to GDF**	80%	90%	-

Table S2. *p*-values among groups.

Domain	Phylum	CD-Control	CD-Probiotic T1	Control-Probiotic T1
Archaea	Euryarchaeota	0.000079	n.s.	0.00009
Bacteria	Actinobacteria	0.0036	n.s.	n.s.
Bacteria	Firmicutes	0.0022	0.0022	n.s.

Table S3. Mean values of relative abundance with standard deviation.

Phylum	CD	Control	Probiotic T1
Euryarchaeota	0.01 ± 0.003	0.4 ± 0.5	0.011 ± 0.02
Actinobacteria	4 ± 3	8 ± 5	6.25 ± 4
Firmicutes	47 ± 15	55 ± 17	54 ± 18

Table S4. *p*-values among groups.

Domain	Phylum	Control-Probiotic T0	Control-Probiotic T1	Probiotic T0-Probiotic T1
Archaea	Euryarchaeota	0.0000195	0.00009	n.s.
Bacteria	Actinobacteria	0.0021	n.s.	n.s.
Bacteria	Firmicutes	0.0003926	n.s.	0.000536
Bacteria	Proteobacteria	0.0031	0.0018	n.s.

Table S5. Mean values of relative abundance with standard deviation.

Phylum	Control	Probiotic T0	Probiotic T1
Euryarchaeota	0.4 ± 0.5	0.018 ± 0.03	0.011 ± 0.02
Actinobacteria	8 ± 5	4.8 ± 3	6.25 ± 4
Firmicutes	55 ± 17	48.2 ± 17	54 ± 18
Proteobacteria	12 ± 6	6.23 ± 5	5.134 ± 4.8

Table S6. *p*-values among groups.

Domain	Phylum	Control-Probiotic T0	Control-Probiotic T1	Probiotic T0-Probiotic T1
Archaea	Euryarchaeota	0.0000195	0.00009	n.s.
Bacteria	Actinobacteria	0.0021	n.s.	n.s.
Bacteria	Firmicutes	0.0003926	n.s.	0.000536
Bacteria	Proteobacteria	0.0031	0.0018	n.s.

Table S7. Mean values of relative abundance with standard deviation.

Family	Control	Probiotic T0	Probiotic T1
Unclass Deltaproteobacteria	0.0001 ± 0.00065	0.02 ± 0.005	0.152 ± 0.0039
Lactobacillaceae	0.028 ± 0.0035	0.005 ± 0.00065	0.019 ± 0.006
Gracilibacteraceae	0.0008 ± 0.00005	0.003 ± 0.00022	0.0123 ± 0.0048
Methanobacteriaceae	0.363 ± 0.1	0.01 ± 0.002	0.0095 ± 0.0008
